# Husbandry Conditions and Welfare State of Pet Chinchillas (*Chinchilla lanigera*) and Caretakers’ Perceptions of Stress and Emotional Closeness to Their Animals

**DOI:** 10.3390/ani14213155

**Published:** 2024-11-03

**Authors:** Elisabeth M. Gilhofer, Denise V. Hebesberger, Susanne Waiblinger, Frank Künzel, Cornelia Rouha-Mülleder, Chiara Mariti, Ines Windschnurer

**Affiliations:** 1 Centre for Animal Nutrition and Welfare, Clinical Department for Farm Animals and Food System Science, University of Veterinary Medicine Vienna (Vetmeduni), Veterinärplatz 1, 1210 Vienna, Austria; lisagilhofer@yahoo.com (E.M.G.); denise.hebesberger@gmx.at (D.V.H.); susanne.waiblinger@vetmeduni.ac.at (S.W.); 2 Clinical Centre for Small Animal Health and Research, Clinical Department for Small Animals and Horses, University of Veterinary Medicine Vienna (Vetmeduni), Veterinärplatz 1, 1210 Vienna, Austria; frank.kuenzel@vetmeduni.ac.at; 3 Animal Welfare Ombudsman Office, Office of the Provincial Government of Upper Austria, Bahnhofsplatz 1, 4021 Linz, Austria; tierschutzombudsstelle@ooe.gv.at; 4 Department of Veterinary Science, University of Pisa, Viale delle Piagge 2, 56124 Pisa, Italy; chiara.mariti@unipi.it

**Keywords:** chinchillas, housing, enrichment, feeding, health, stress, behavior, human–animal relationship, welfare

## Abstract

For pet chinchillas, limited data are available on husbandry and the human–animal relationship despite their impact on health, behavior, and welfare. We conducted an online survey with pet chinchilla caretakers (*n* = 336), targeting husbandry practices, health, behavioral indicators of welfare, and human–chinchilla relationships. We further investigated associations between caretakers’ perceptions of stress in their chinchillas and emotional closeness to their animals. Basic needs such as keeping with conspecifics, constant access to water and hay, or offering dust baths were mostly fulfilled. Potential welfare issues included individual keeping (14.3%), undersized cages/enclosures (reported by 27.6% of the Austrian respondents), and suffering from a disease (14.7%). Behavioral indicators of good welfare, such as playing and cuddling with conspecifics, were observed several times per day by 40.9% and 87.9% of the respondents, respectively. Repetitive and unwanted behaviors were less common (fur biting, for instance, occurred ‘never’ in 82.9%). Caretakers rated their animals as generally more stressed if the animal was ill and more often showed fearful behavior toward them. Caretakers feeling closer to their animals spent more time engaging with them. Correct identification of this kind of association could be used as guidance for recommendations to improve chinchilla welfare at home and in the veterinary setting.

## Simple Summary

For pet chinchillas, limited data are available on husbandry and the human–animal relationship despite its impact on their health, behavior, and welfare. We conducted an online survey addressing chinchilla husbandry practices, health, behavioral indicators of welfare, and the human–animal relationship. We also examined associations with caretakers’ perceptions of stress in their chinchillas and emotional closeness to their animals. Basic needs such as keeping with conspecifics, constant access to water and hay, or offering dust baths were mostly fulfilled. Potential welfare issues included individual keeping, undersized cages/enclosures, and suffering from a disease. Behavioral indicators of good welfare, such as affiliative behaviors, were observed by the majority of caretakers several times a day. Repetitive and unwanted behaviors were less commonly reported (fur biting, for instance, occurred ‘never’ in most cases). Caretakers rated their animals as generally more stressed if the animal was ill and more often showed fearful behavior toward the caretaker. Caretakers feeling closer to their animals spent more time engaging with them. Identifying such associations can help to formulate recommendations to improve pet chinchillas’ welfare.

## 1. Introduction

The long-tailed chinchilla (*Chinchilla lanigera*) is a crepuscular and nocturnal, exclusively herbivorous rodent whose wild form lives in colonies of up to several hundred animals in its natural habitats in South America [1,2]. It was first domesticated in the 1920s and has since been kept worldwide for fur production, as a laboratory animal, and as a pet [1,3]. The husbandry conditions of chinchillas are crucial for their health, behavior, and welfare. Although chinchillas have lived in human care for around 100 years, limited to no data based on scientific studies are available on their husbandry (such as housing types, enrichment, feeding, care), behavior, and the human–animal relationship regarding chinchillas kept as companion animals [4,5,6].

As a part of the large-scale “Exopet Study”, which collected information on the keeping of exotic animals in Germany, 105 online questionnaires from chinchilla caretakers were evaluated [5]. The questions targeting husbandry conditions of chinchillas were rather general, except for a few more detailed questions concerning, for instance, the availability of dust baths. No questions were asked about the human–animal relationship or the behavior of chinchillas. A questionnaire study on the husbandry of pet chinchillas in Italy [6] collected more extensive information, but further research on the husbandry, behavior, health, and human–animal relationship in pet chinchillas is needed to get a more comprehensive insight into the welfare state of pet chinchillas. Moreover, the findings in Italy [6] might not necessarily represent current husbandry practices in other countries. A significant number of studies focused on chinchillas raised for the fur industry, in particular on the issue of fur biting [7,8,9,10,11]. However, the conditions in which they are kept on commercial fur farms cannot be transferred to the conditions under which they are kept as pets.

The Five Domains model [12,13] outlines essential requirements for animal husbandry, such as the provision of adequate nutrition, a suitable physical environment, health (care), opportunities for natural behaviors and positive human–animal interactions, and consideration for animals’ mental well-being. In order to identify potential welfare problems and subsequently make targeted changes, it is essential to survey the current husbandry conditions of chinchillas kept as companion animals and to compare them to their natural behavior and species-specific needs.

As social animals, chinchillas should not be kept alone [1,3,14] because social isolation can cause stress [15]. According to chinchilla specialists, they can be kept in pairs, in female/male/polygamous groups or a sibling group—ideally two to six animals together [2,14,15]. As chinchillas are very active and like to climb, they should be kept in a sufficiently large cage with several levels and appropriate heights [3,15,16]. Cage size and equipment can have an influence on the behavior of the animals, including their activity and behaviors toward humans [17]. Chinchillas are generally shy animals, sensitive to sudden movements, bright light, and sudden noise, and quickly flee to a narrow, dark place [15]. Accordingly, hiding places such as tubes or tunnels should be offered [3]. Opportunities to climb and jump (e.g., raised areas) are important [15], as is an appropriate running area, since a lack of roaming possibilities can cause behavioral problems [4]. Sleeping places should be available in sufficient numbers so that the animals can rest both alone and in company in line with their social nature [15]. Furthermore, dust baths are essential for the welfare of chinchillas and should be provided several times a week or daily [2,3,18]. Dust bathing not only helps chinchillas to maintain and clean their coats, but also to reduce stress [19]. Dust or sand specially made for chinchillas from specialist shops or volcanic ash should be used, as opposed to conventional sand from the hardware store or the beach [2,18].

Also, the climate in their housing environments must be considered. In their natural habitats, chinchillas live under quite harsh conditions in barren, rocky, sandy, and windy environments with low rainfall and drastic temperature changes (reviewed by [20]). Chinchillas can cope well with the cold in a dry, draft-free place [1,2]. For instance, they use rock crevices as shelter and protection from rain and temperature changes [16]. However, high temperatures and high humidity are problematic and, in the worst case, can lead to heat stroke [18,21].

Proper feeding and sufficient water intake are crucial factors in preventing, for example, dental problems, diarrhea, or the development of urinary stones [22,23,24]. The main component of chinchilla food should be hay in order to come as close as possible to the natural food available to wild chinchillas [23,25]. Fresh leaves and herbs should be offered regularly, while concentrate food should only be fed in limited quantities [3,15]. (Dried) fruits or nuts should be avoided as they can cause medical problems [26,27]. Water should always be freely available [15].

The importance of the housing environment is also reflected in the occurrence of husbandry-related diseases and injuries. For example, running wheels with open surfaces pose a risk of injury [28,29]. Since plastic objects can be eaten and swallowed, they should be avoided in areas chinchillas can access without supervision [16,21]. Dust baths that exceed normal duration and frequency can cause eye irritation and subsequent conjunctivitis, as well as irritation of the respiratory tract, so some experts recommend removing the bath after use [2,3,30]. Furthermore, cleaning and health care routines are important. Regular cleaning of the enclosure is essential to remove or eliminate potential pathogens [31,32,33]. The dust bath should be cleaned daily and the dust replaced accordingly [3,15]. Regular checks carried out by the animal caretakers or, if necessary, veterinarians, such as teeth checks, also help to maintain and promote animal health [34].

Unfavorable housing conditions, such as poorly equipped cages or lack of social contact, can cause abnormal behavior [7], indicating impaired welfare [35]. Stressful husbandry conditions can, on the one hand, lead to physical changes such as being overweight or underweight, and, on the other hand, to behavioral problems, such as increased levels of aggression, bar biting, or running up and down between certain parts of the cage (potential stereotypies) [15]. The most frequently described behavioral problem in chinchillas is fur biting, mainly due to the prominence of the problem as well as the economic impact on fur production [7,8,9,36,37]. Since the animals are nocturnal [17], other behavioral abnormalities such as gnawing on cage bars or backflips can often remain undetected for a long time [7].

It is vital to consider the species-specific needs and behavior of chinchillas not only when designing their housing environment, but also when handling them. People can trigger a stress reaction in chinchillas, especially if the animals had few or bad experiences with them [15]. Lifting, in particular, can induce fear in these prey animals [15]. If the animals start struggling to escape, they can be dropped accidentally and injured seriously [15]. Due to their fearful nature and stress perceptiveness, chinchillas are not suitable as pets for small children [14,15,16]. When frightened by people, conspecifics, or other animals, chinchillas can make shrill noises, spray urine, or try to defend themselves by biting [3,15,38]. This can be perceived as problem behavior that may deteriorate the human–animal relationship. The satisfaction of caretakers with their pets can decrease when the animals show such unwanted behavior [4]. Unwanted or problem behavior of pets can result in the relinquishment of the animals to an animal shelter. A better human–animal relationship is generally linked with more frequent positive interactions with the animals and often better housing conditions (as shown, for instance, in cats [39]). Aspects of the human–animal relationship, such as the emotional closeness of caretakers to their animals, can be influenced by household characteristics such as the presence of children in the household [40,41].

Regarding human–animal interactions, it is always important to consider the animals’ degree of fear or confidence in humans [42]. If animals are not used to close contact, it might cause fear. Thus, it is vital that caretakers give their animals control and choice over how and when to interact with their caretakers [43]. Overall, caretakers who do not pay close attention to the animals’ needs, behavior, and potential signs of fear and stress may not recognize such signs or react too late [44].

One aim of this study was to give an insight into the husbandry conditions of pet chinchillas in German-speaking countries (including housing conditions, group compositions, feeding routine, and health care), their state of health, and the occurrence of behaviors that could reflect good or impaired welfare by conducting an online survey among chinchilla caretakers. Another objective was to explore the human–animal relationship, looking into the emotional closeness of caretakers toward their chinchillas and into human–animal interactions. Furthermore, we were interested in associations between the emotional closeness of caretakers and their animals and the caretakers’ perceptions of stress in their chinchillas. We assumed that caretakers would perceive their chinchillas as generally less stressed if husbandry conditions better met the behavioral needs of the chinchillas (such as more roaming possibilities and dust bath access), absence of illness and disliked behavior, if they spent more time engaging with their chinchillas (hand-feeding, playing, training, observing), as well as, in case of lower occurrence of repetitive and agonistic behaviors, fewer signs of fear of the caretaker and higher occurrence of affiliative behaviors. Furthermore, we expected that stronger attachment to the chinchillas (a closer emotional bond) would relate to better housing conditions, the absence of younger children in the household, no reports of disliked behavior, less agonistic and fearful behavior in the caretakers’ presence, and more frequent affiliative behaviors, as well as more frequent health checks and care measures and more positive human–animal interactions.

## 2. Materials and Methods

### 2.1. Questionnaire Development

A questionnaire study aimed at chinchilla caretakers in German-speaking countries (Austria, Germany, Switzerland, and Luxembourg) was conducted. Questionnaires that were formerly developed to assess the husbandry and welfare of ferrets [45] and chinchillas kept in Italy [6] served as the starting point for the formulation of the questionnaire. Questions targeting the human–chinchilla relationship were based on questionnaires assessing human–animal interactions in other species (fattening bulls [46], ferrets [47]) and a scale assessing emotional closeness to pets (Comfort from Companion Animal Scale by [48]). The Comfort from Companion Animal Scale analyzes the attachment of animal caretakers to their animals as well as the self-perceived emotional welfare they gain from animal ownership.

The first version of the chinchilla questionnaire was checked by four veterinarians (two working in the field of animal welfare science, one specialist in internal veterinary medicine, and one ombudsperson for animal welfare), two students of veterinary medicine, and finally by a chinchilla caretaker. Unclear or ambiguous questionnaire content was revised according to the feedback from these people.

### 2.2. Conditions for Participation and Questionnaire Structure

At the beginning of the questionnaire, participants were briefed in an informed consent section about the content, that they could quit this anonymous survey at any time, and that there were no mandatory answers. All participants agreed to these terms by starting the survey.

The prerequisite for taking part in the survey was to own at least one chinchilla at the time of answering; participants who did not own a chinchilla at that time were disqualified. Participants were also asked to answer the questionnaire only if they were the main caretakers of the animals, and, in the case of minors, in the presence of adults.

In order to obtain data that are as objective as possible and to prevent participants from choosing their favorite animal when keeping several chinchillas, they were asked to answer various questions only in relation to one of their chinchillas, specifically the animal whose name came first alphabetically (called “A-animal” cf. [49]). We refer to it in the manuscript as the “focus animal”.

The questionnaire comprised 55 to 62 questions with sub-questions, depending on husbandry conditions, because some questions targeted specific situations (including reasons for individual housing or behavior toward conspecifics). The questionnaire contained single and multiple-choice questions, Likert scales, and yes/no questions. Participants could also enter answers or explanations for various questions in comment fields. A description of the questionnaire sections and questions relevant to this publication is provided in Table 1.

### 2.3. Online Survey and Recruitment of Participants

The survey was available between May and September 2021 via the SurveyMonkey^®^ online platform and was accessible via a link. It was posted on the Facebook page of the University of Veterinary Medicine, Vienna, and advertised in chinchilla groups by the first author. In addition, the first author wrote to breeders and operators of chinchilla adoption websites via email or their respective websites to advertise the survey. In order to increase the willingness to participate, a raffle with non-cash prizes (including books and chinchilla food) was offered at the end of the survey.

### 2.4. Response Rate

Altogether, 467 participants took part in the study; 129 questionnaires were excluded from the analyses because participation ended after the first general questions about chinchilla husbandry. The prerequisite for inclusion in the data analysis was that at least question eleven (How many chinchillas live in the focus animal’s main living area [=permanently accessible area]?) was answered. After checking the plausibility of the answers provided, two more questionnaires were excluded because they showed significant similarities to other questionnaires and were thus considered potential duplicates. Ultimately, 336 questionnaires were included in the analysis if data had been provided for the respective questions.

### 2.5. Data Analysis

Since no question had to be answered obligatorily and some questions targeted specific situations (such as behavior toward conspecifics in the case of pair or group housing), the sample size varies across the results presented. Therefore, the respective sample sizes (total number of available replies) are reported with each result. Answers given in comment fields were analyzed and summarized by conducting a qualitative content analysis.

In four out of a total of eleven questions with a drop-down menu with an infinite maximum (such as “over 15 years”, “more than ten floors”), some respondents chose these maximum values. The number of responses with this upper limit is given separately in the results section. This was the case for the question targeting experience in keeping chinchillas (maximum value applicable “over 15 years”), the number of chinchillas kept currently (“over 100 animals”), as well as the height of and the number of floors of the enclosure of the focus animal (“over 300 cm” or “more than ten floors”).

After plausibility checks, five reports of the commercial cage exceeding a ground floor of 6 m^2^ and three reports of self-built enclosures exceeding 12 m^2^ (including a floor space of up to 101.5 m^2^ in some cases) were excluded from the results. It was assumed that the question was not properly understood by the respective respondents because the measures seemed implausible. For the same reason, three reports of ‘chinchilla rooms’ smaller than 2 m^2^ and seven reports of ‘chinchilla rooms’ with contradictory information about the size of the main living area were excluded.

Statistical analyses were performed using SPSS (IBM SPSS Statistics for Windows, version 29.0, IBM Corp., Armonk, NY, USA). In terms of descriptive statistics, mean, standard deviation, minimum, maximum, median, frequencies, and percentages were calculated.

Principal component analysis (PCA) was used to summarize (health) care measures, human–animal interactions, and chinchilla behaviors (social, unwanted, and repetitive behavior, and behaviors toward the caretaker) to a smaller number of components. A PCA was also run for the eleven Comfort from Companion Animal Scale items to check if we would obtain sufficiently high component loadings (>0.4) with our chinchilla data in order to use the scale originally validated for cats and dogs [48].

In sum, six PCAs were calculated. Data suitability for PCA was verified by means of the Kaiser–Meyer–Olkin Measure of Sampling Adequacy and Bartlett’s test of sphericity. The Kaiser–Meyer–Olkin criterion had to be at least 0.5, and Bartlett’s test of sphericity had to be significant (*p* < 0.05) [50]. We performed Varimax rotations on component solutions in case of more than a single component to help simplify the interpretation [51].

Regarding component loadings of items, the threshold was set at 0.4 and greater for an item to be included in the respective component. This limit was chosen because loadings above 0.3–0.4 are commonly found in the behavioral and psychological literature [52]. In case an item loaded on more components, it had to have a loading higher than 0.6 on the component it would be included in, and the loading(s) on the other component(s) had to be below 0.4. PCAs were recalculated after excluding variables with loadings below 0.3 and variables that loaded on more components without loadings higher than 0.6 on one and below 0.4 on the other(s).

We determined the number of components for each PCA based on the Kaiser criterion (Eigenvalue of at least 1.0) and confirmed it by means of screen plot inspection and component interpretability [51]. When labeling the components, we considered the semantic content of the items. Finally, to allow comparability for each component, we calculated mean scores to have the averages on the same Likert scale as the original questionnaire items. For the Comfort of Companion Animal Scale, the CCAS score was calculated according to the literature [48], summarizing the scores of the eleven questions. With eleven questions on a 4-point scale, a minimum score of eleven and a maximum score of 44 were possible.

In order to evaluate associations between husbandry conditions, human–animal interactions, and animal behavior with the caretakers’ general perception of stress in their chinchillas and the emotional closeness to the animals (CCAS score), two linear regression models were calculated. Independent variables for the stress perception model were five variables related to housing conditions that could relate to stress in chinchillas (frequency of dust bath access, duration of access and frequency of access to a running area, size of the main living area, group size). Moreover, we included in the model the daily time the caretaker spends with the focus animal, the absence or presence of illness and disliked behavior, as well as frequency of repetitive, affiliative, agonistic (both toward conspecifics and the caretaker), and behaviors indicative of caretaker fear.

As independent variables in the emotional closeness model (CCAS score), we selected the presence or absence of children and three housing condition variables that might relate to emotional closeness to the chinchillas (duration and frequency of access to a running area and frequency of dust bath access). In addition, we included the daily time the caretaker spends with the focus animal and further human–animal interaction variables (such as frequency of lifting the animals and carrying them around) as well as (health) care measures summarized by means of PCA. In terms of chinchilla behavior, the presence or absence of disliked behavior and the frequency of affiliative, agonistic (both toward conspecifics and the caretaker), and fearful behaviors in the presence of the caretaker were entered into the model. Contrary to the perceived stress level model, we decided against including repetitive behaviors in the CCAS score model because we thought them less likely to relate to the degree of attachment to the chinchilla.

Due to the exploratory nature of our study and the cross-sectional study design, only conclusions on associations, not on causal relationships, are possible. The independent variables are not interpreted as predictors in the regression models, but we looked at significant associations with perceived stress levels and emotional closeness (CCAS score). We deliberately decided to use regression models to avoid running multiple univariate analyses.

Normal distribution of residuals was inspected graphically by means of a P-P plot, and the homoscedasticity assumption was inspected by plotting the standardized residuals against the standardized predicted values resulting from the model. Regarding multicollinearity, we checked the variance inflation factors (VIF values had to be <4.0). Both linear regression models met the model assumptions. We refer to results with *p* ≤ 0.05 as significant and to results with *p* > 0.05 ≤ 0.1 as “trends”.

## 3. Results

### 3.1. Participants and Household Characteristics

The country of origin of the participants was predominantly Germany, accounting for 83.8% (Table 2). The majority of participants were female (93.0%, male: 6.6% (20), diverse: 0.3% (1), *n* = 301) and kept the chinchillas for themselves (96.1%, *n* = 336). More than a quarter (26.0%, *n* = 87) had kept chinchillas for more than 15 years. Most frequently, two chinchillas were kept at the time of the survey (48.2%, *n* = 336), two caretakers (0.6%) stated that they kept over 100 chinchillas. Participants stated that they spent an average of 2.0 ± 1.3 h per day on all their chinchillas (focus animal and others; min–max= 0.25–12.0 h/day; median= 2 h/day; *n* = 331). This included providing food, cleaning the cage/enclosure, care measures, and direct interactions such as training, playing, and stroking. The majority of households comprised two to three people (69.7%, *n* = 300), followed by one-person households (16.3%). For further details, see Table 2.

### 3.2. Focus Animals and Group Composition

Slightly more than half of the focus animals were male (Table 3; 51.8%, *n* = 332) and most of the animals were intact (males: 66.8%, females: 95.0%, *n* = 332). The age of the focus animals ranged from four months to 24 years (mean ± SD: 6.6 ± 5.2 years, *n* = 317). The most common origin was acquisition by private individuals (28.4%), 21.5% had gotten their focus animal from an animal shelter or an animal welfare organization (*n* = 335), another 20.3% of the animals came from hobby breeding, and 12.8% from professional breeding. The focus animals had lived with the participants between under four weeks and 23 years (4.5 ± 5.0 years, *n* = 330). Within the last eight weeks before participating in the survey, the group in which the focus animal lives had increased by 6.9% and decreased by 7.3% for all cases (*n* = 331). The group composition was most frequently given as a mixed-sex pair (Table 3: 28.1%, *n* = 258) followed by a male pair (22.4%). However, 14.3% of the participants (*n* = 336) stated that they kept their focus animal individually, whereas 54.3% of these participants (*n* = 46) stated that socialization with another chinchilla did not work as a reason for keeping them alone (multiple answers possible). In 22 cases, the partner animal had died, three animals had already been adopted from single keeping, and one male animal lived alone only temporarily because it was kept separately after castration. Due to current illness, three animals were kept without conspecifics, and two animals were kept individually but had temporary contact with other chinchillas (*n* = 46). Five participants further explained that socialization of the focus animal was planned or was currently underway.

A total of 34.3% of the focus animals had contact with other species, including cats and dogs (for details see Table 3).

### 3.3. Husbandry

#### 3.3.1. Housing Type and Additional Running Area

The majority of focus animals (70.6%) were housed in self-built enclosures (e.g., restructured wardrobe) or commercially available cages, both with temporary access to an additional running area (self-built: 58.8%, commercial: 11.8%, *n* = 330). A total of 10.9% of the focus animals were kept in their own ‘chinchilla room’, of which 4.5% were also able to move around in other living areas at times. Another 8.8% of the focus animals lived in self-built enclosures or commercially available cages without an additional running area, and 8.1% were kept in self-built enclosures or commercially available cages with constant access to an additional running area. The entire apartment was available to three focus animals (0.9%) at all times (*n* = 330). Two other people indicated an unspecified other housing situation.

A total of 38.0% of participants gave their focus animal daily access to the occasional running area, 23.4% several times a week, and 10.3% several times a day (*n* = 321). If free roaming was allowed, it lasted between less than half an hour and up to 18 h. The majority of focus animals (19.9%) had access to the running area for an average of one hour (*n* = 241), followed by two hours (18.7%), and an hour and a half (18.3%).

#### 3.3.2. Dimensions of the Main Living Area

The average floor area of the main living area (commercially available cages or self-built enclosures) for the focus animal (and conspecifics, in case of pair or group housing) was 1.9 m^2^ ± 1.7 m^2^ (min: 0.25 m^2^, max: 12 m^2^, *n* = 271). The smallest specified cage size was 0.25 m^2^ in two cases, with one person indicating that it was a quarantine cage. The average floor area of the ‘chinchilla room’ was 12.0 m^2^ ± 6.3 m^2^ (min 4.0 m^2^, max: 32 m^2^, *n* = 23). Two participants stated that they kept their focus animal in the entire apartment of 60 m^2^ or 77 m^2^; the third participant did not provide a size.

Half of the cages and enclosures (50.0%) were between 161 cm and 200 cm in height (*n* = 328). The height ranged from 41–60 cm to 281–300 cm. One person reported a height of over 300 cm. Most focus animals (73.6%, *n* = 303) had between three and six floors in their cages/enclosures. One person only held their focus animal at ground level, without an elevated platform; an additional 19 participants stated that the cages/enclosures had more than ten floors, including platforms.

#### 3.3.3. Furnishings and Enrichment

A total of 32.0% of participants stated that they offered at least one running wheel (*n* = 297) and 31.8% reported offering at least one running plate in the main living area (*n* = 289). Most of the running wheels were made of metal (35.0%), 20.1% of wood, and 0.6% were made of plastic (*n* = 309). Regarding the tread, 42.6% had closed running wheels without openings and 7.3% had open running wheels with cross braces (*n* = 289). Dust baths were available to 99.7% of focus animals (*n* = 333). One person (0.3%) stated that the animal did not have access to the dust bath, 0.9% offered it several times a week, 1.8% daily, and 0.3% several times per day. The majority of focus animals (96.7%, *n* = 333) had constant dust bath access. Most participants (*n* = 331) used attapulgus (47.4%) or special chinchilla bath sand (45.0%) in the dust bath.

Table 4 provides an overview of how often participants reportedly offered further furnishings and enrichment such as fixed roots, branches, sleeping houses, tunnels, or boards at different heights.

#### 3.3.4. Feeding and Water Provision

Food bowls (95.2%) and water sources (98.2%) were constantly available to most focus animals (*n* = 334). Two participants stated that they only provided water sources several times a week. Activity feeding enrichment such as a food tree or a food ball was constantly offered to 39.3% of the animals (*n* = 326). Hay was constantly provided by 90.1% (*n* = 333) of the caretakers and they were never fed dairy products (*n* = 330). A detailed overview of nutrition and water provision is presented in Table 5.

#### 3.3.5. Climate Control and Cleaning of the Housing Environment

In the room containing the focus animal’s enclosure, 14.5% of participants reported using a fan, 35.0% had air conditioning, and 13.3% had a dehumidifier (*n* = 331). A hygrometer was used in 22.7% and a thermometer in 51.1% of cases. Almost a third of the participants (30.5%) stated that they did not use any systems for monitoring and controlling the indoor climate (*n* = 331, multiple answers possible).

The entire accommodation was cleaned once a week by 36.3% of caretakers (*n* = 328). For further details, see Table 6.

### 3.4. Health and (Health) Care Measures

A total of 14.4% of participants stated that their focus animal was currently acutely or chronically ill (*n* = 327). The most frequently reported current diseases diagnosed by veterinarians (*n* = 45) concerned teeth (42.2%), followed by diarrhea (6.7%), neurological abnormalities (6.7%), tumors (6.7%), broken bones (4.4%), breathing problems (4.4%), skin problems (4.4%), and bite injuries (2.2%). Lameness, urolithiasis, hair loss, pododermatitis, hair rings, abscesses, or parasites were not reported. In addition, six participants indicated heart problems in their focus animal using a text field and three focus animals suffered from eye diseases. Some of the “illnesses” specified in the text field are more likely impairments, including blindness, which was reported four times.

A total of 47.3% visited vets with their focus animal at least once a year, 24.2% less than once a year, and 12.3% stated that their focus animal had never been taken to a veterinarian (*n* = 326). Using a text field, 16.3% of participants stated that they only presented the focus animal to veterinarians in the event of illness.

The most frequently performed care measure was control of the incisors, which was performed by 92.4% of caretakers (*n* = 327). Details on the frequency of (health) care measures carried out are provided in Table 6.

Following principal component analysis (PCA), (health) care measures were summarized into two components that explained a total variance of 70.9%. The first component comprised checking the ears, anal region, and incisors, and was labeled ‘health checks’. Its component loadings ranged from 0.84 to 0.88 (for details see Supplementary Material, Table S1). The second component comprised three (health) care measures (cleaning of the eye area/nasal area, and fur care, e.g., brushing), with loadings ranging from 0.59 to 0.90. It was named ‘cleaning & fur care’.

### 3.5. Human–Animal Relationship

#### 3.5.1. Emotional Closeness to the Animals

Attachment of the participants to their focus animal was assessed by means of the Comfort from Companion Animal Scale (CCAS) [48]. A minimum score of eleven and a maximum score of 44 are possible, with higher scores indicating a closer attachment. The mean CCAS score was 35.9 ± 5.5 and ranged from 15 to 44 (median: 35.0, *n* = 299, see Supplementary Material, Figure S1). Regarding the PCA of the items from the Comfort from Companion Animal Scale [48], we forced a single-component solution to be consistent with the original scale. This single-component solution explained 49.4% of the variance, with component loadings ranging from 0.42 to 0.82 (see Supplementary Material, Table S2).

#### 3.5.2. Human–Animal Interactions

**Figure 1:**
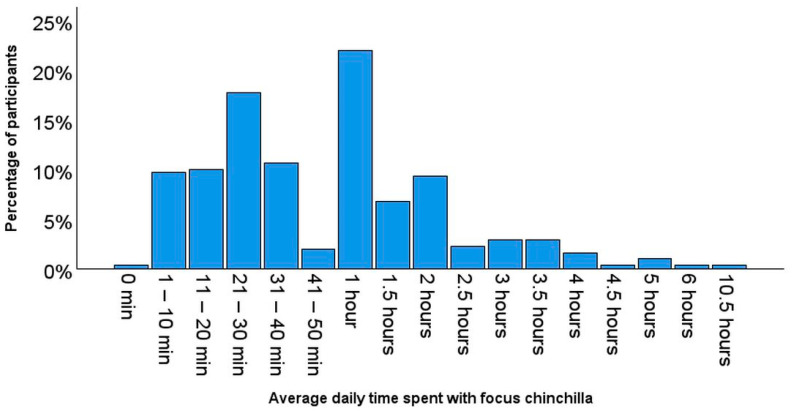
Bar chart demonstrating the average daily time participants (*n* = 310) reported engaging with their focus animals.

The average time period most frequently spent by participants with their focus animal (“including hand-feeding, stroking, playing, training, observing, etc.“, according to the question referred to in the survey) was one hour per day (Figure 1: 22.0%, *n* = 310). One participant reported not engaging with the focus animal while another reported 10.5 h spent with the focus chinchilla.

The majority of participants reported observing their chinchillas (75.8%, *n* = 310) and talking to them (79.5%, *n* = 312) several times per day. Further details about the frequency of various human–animal interactions are provided in Table 7.

For the principal component analysis, the categories of ‘never’ were summarized. Principal component analysis of human–animal interactions revealed three components that explained a total variance of 70.2%. The first component comprised three items (talking to, observing, and hand-feeding) and was labeled ‘observe talk hand-feed’. Its component loadings ranged from 0.64 to 0.85. The second and the third components had two items each and were labeled ‘lifting and carrying around’ (comprising lifting up the chinchilla and carrying it around) and ‘training’ (including clicker and target training). Component loadings were high, with 0.90 and 0.90 for ‘lifting and carrying around’, and 0.77 and 0.86 for the items included in ‘training’ (for details see Supplementary Material, Table S3).

### 3.6. Behavior of the Focus Animal

A total of 67.8% of participants stated that their focus animal became hand-tamed through habituation, 56.5% used food for this purpose, and 16.5% claimed that they had adopted the focus animal already hand-tamed (*n* = 255, multiple answers possible). Another 2.0% of the caretakers had worked with clicker training in order to tame their chinchillas.

With regard to repetitive behavior, running up and down or jumping at a specific cage/enclosure location or between two specific cage/enclosure locations was reported most frequently (Table 8: 30.1%, *n* = 306), followed by biting or shaking the enclosure bars (25.1%, *n* = 307), and biting their own fur (17.1%, *n* = 304). One-third of the participants (34.5%, *n* = 281) stated that the focus animal tried to block or drive conspecifics away from food. In terms of reactions toward the caretaker, 9.4% (*n* = 308) of the focus animals ran away from their caretakers or avoided them several times a day.

For the frequency of occurrence of specific behaviors of the focus animal toward conspecifics and the caretaker as well as repetitive and unwanted behaviors, see Table 8.

By running a PCA, social behaviors were summarized into two components explaining 53.2% of the total variance. The first component comprised the four agonistic behaviors, i.e., chasing or fighting with conspecifics, biting conspecifics, driving away/blocking conspecifics from food, as well as spraying urine at conspecifics, and was thus labeled ‘agonistic behavior’. The component loadings ranged from 0.62 to 0.76 (for details see Supplementary Material, Table S4). The second component comprised affiliative behavior only and was therefore named that way (two behaviors: cuddling/lying snuggled up with conspecifics and simultaneous peaceful eating with conspecifics, with loadings ranging from 0.58 to 0.85).

Following PCA, negative reactions to caretakers were summarized into two components that explained 68.0% of the total variance. The first component comprised the items freezing/startling, avoiding or running away, and making noises (such as chattering of teeth, hissing, screaming, and single shrill sounds), with component loadings ranging from 0.66 to 0.81. It was labeled ‘fearful behavior in presence of caretaker’. The second was the single-item component ‘agonistic behavior toward caretaker’ (frequency of negative interactions with caretaker [aggression, biting, urine spraying]) with a high component loading of 0.98 (For further details see Supplementary Material, Table S5).

Principal component analysis (PCA) of unwanted and repetitive behaviors resulted in a three-component solution with two items each. The total variance explained was 65.8%. The first component comprised the items biting/nibbling fur of conspecifics and biting/nibbling own fur and was labeled ‘fur biting’. Component loadings were high, with 0.86 and 0.89, respectively (for details see Supplementary Material Table S6). The second component, which was labeled ‘repetitive behaviors/escape attempts’, comprised the behaviors bar biting/bar shaking and running up and down or jumping at a certain place in the cage/enclosure or between two places. The component loadings were 0.77 each. The third component comprised the items biting into own tail and backflips, with loadings of 0.75 and 0.70. It was referred to as ‘other abnormal repetitive behavior’.

### 3.7. Perceived Stress Level of the Focus Animal

**Figure 2:**
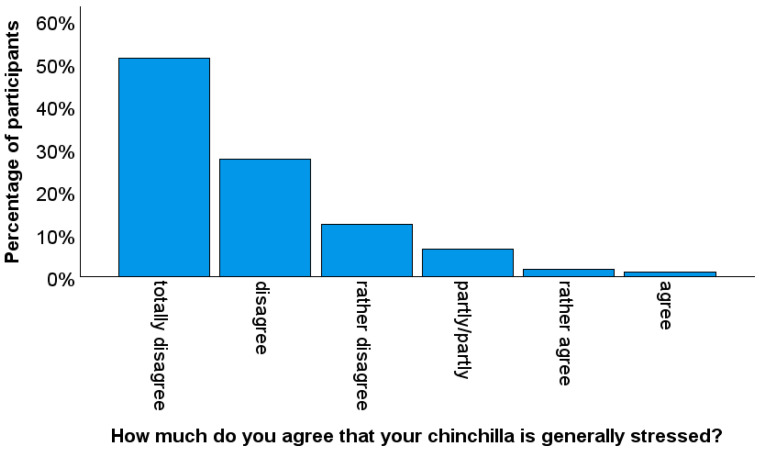
Bar chart representing the degree of agreement with the focus animal generally being stressed, assessed on a 7-point scale from ‘totally disagree’ to ‘totally agree’ (*n* = 310).

Just over half (51.3%) of the participants stated that they completely disagreed with the statement that their focus animal was generally stressed (*n* = 310). None of the participants fully agreed with the statement. Details are shown in Figure 2.

### 3.8. Associations with the Perceived Stress Level of the Focus Animal

The linear regression model run to identify associations with stress caretakers perceive in their focus animals is presented in Table 9. Perceived stress levels were significantly higher if the focus animal was currently ill and showed fearful behavior toward the caretaker more often. Moreover, perceived stress levels tended to be higher if a lower number of chinchillas were kept with the focus animal, when affiliative behaviors were observed less often, and in the case of more frequent fur biting.

### 3.9. Associations with the Emotional Closeness to the Focus Animals

An overview of the linear regression model run to identify associations with caretakers’ emotional closeness to the focus animals, assessed by means of the Comfort from Companion Animal Scale (CCAS), is provided in Table 9. Time spent daily with the focus animal was a significant predictor of the CCAS score of participants, while the frequency of lifting the chinchilla and carrying it around and the presence of children in the household tended to correlate with the CCAS score. Participants who had a closer bond with the chinchillas spent more time engaging with them and tended to lift the chinchillas up and carry them around more frequently. Moreover, participants tended to feel closer to their animals when children were not present in the household.

## 4. Discussion

This questionnaire study provided comprehensive insights into housing conditions, group composition, feeding routines, (health) care measures, health and behavior of pet chinchillas, as well as the human–animal relationship. Moreover, associations between husbandry conditions, human–animal interactions, and animal behavior and emotional closeness to the animals (CCAS score by [48]) and caretakers’ perceptions of stress in their chinchillas were also presented.

### 4.1. Participants and Household Characteristics

There was a predominance of female participants (93.0%), which is comparable to studies conducted on pet chinchillas in Germany (92.2%) [5] and Italy (84.8%) [6] and was also observed in other surveys targeting husbandry and human–animal relationships with other companion animals such as dogs, cats, guinea pigs, rabbits, and ferrets [6,39,45,49,53,54]. Women generally exhibit higher levels of concern and empathy for animals compared to men [55,56,57], which may make them more likely to participate in surveys related to animal welfare and the human–animal relationship. Almost half of participants (49.4%) were under 30 years old, similar to the chinchilla studies from Germany (52.2%) [5] and Italy (58.9%) [6], probably also resulting from the use of an online questionnaire for data collection. The most frequently indicated purpose for keeping chinchillas was as pets. Although children and/or young people lived in almost one-third of the households, only a small proportion of participants stated that they kept chinchillas as pets for their kids. This suggests that most caretakers seem to be aware that chinchillas are not suitable pets for (younger) children [15,16].

### 4.2. Focus Animals and Group Composition

The chinchillas’ age ranged from four months to 24 years. Compared to the Italian study [6], which found an age range of between less than half a year and up to 15 years, the stated maximum age appears high; nine (2.7%) focus animals were 18 years or older. Experts specify the life expectancy for pet chinchillas spans from 8 to over 20 years [2,16,58].

Most focus animals lived in their main living area with conspecifics, and the group sizes had remained largely stable in the last eight weeks ahead of the survey. Most frequently (43.1%), the focus chinchillas were kept in mixed-sex pairs or all-male pairs or groups, as recommended by experts [15]. However, one in seven focus animals (14.3%) in this study was kept individually, which seems high compared to the studies conducted in Italy [6] and Germany [5], where 11.2% and 3.9% of chinchillas, respectively, were kept individually. The reasons given, especially failed socialization and death of the partner animal, suggest that most animal caretakers were trying to keep their focus animals together with conspecifics (again) and that it was largely not an intentional case of individual-keeping due to lack of knowledge. Nevertheless, the keeping of social animals such as chinchillas without conspecifics can affect their welfare. However, individual temperaments are important to consider when grouping animals. Distress due to social incompatibility can be a significant welfare issue, as can social stress due to group instability. Pair or group housing should always be attempted unless an animal is genuinely and repeatedly socially incompatible or requires separation for health reasons, such as recovery from surgery or to prevent the spread of contagious diseases.

The presence of a conspecific has a positive influence on rodents, helping them to react more resiliently to stressful situations [59]. Therefore, the question of why socialization attempts are not successful should be clarified in a follow-up study, ideally combined with educating animal caretakers about possible reasons, such as suboptimal gender composition [15,60], to prevent solitary housing in the future.

### 4.3. Husbandry

The cages or enclosures of focus animals in the present studies varied between sizes of 0.25 m^2^ to 12 m^2^. According to experts, cages and enclosures for keeping chinchillas should be as large as possible [15] or at least 2 × 2 × 1 m [16]. Under Austrian law [61], the floor area for two chinchillas must be at least 0.96 m^2^; in Switzerland, it should cover at least 0.5 m^2^ [62]. In Germany, there are no minimum legal requirements but only non-binding guidelines that recommend a minimum size of 2 m^2^ for keeping two chinchillas [58]. To the best of the authors’ knowledge, no legal requirements exist in Luxembourg.

Based on the information provided by participants, 8 (27.6%) out of 30 caretakers in Austria and 146 (70.9%) out of 206 caretakers in Germany seemed to house their chinchillas in insufficiently sized cages or enclosures with dimensions contrary to respective (legal) requirements; this did not apply to any participants from Switzerland. Based on expert opinion, across all four countries (Austria, Germany, Switzerland, and Luxembourg) from which the participants came, a total of 90.4% of the cages and enclosures appear to be too small. The study conducted in Germany [5] found that 62.5% of the chinchillas’ main living areas were too small, which is in line with our findings. An examination of German pet stores [29] showed that none of the offered cages was big enough. On the one hand, this illustrates the need for legal requirements and, on the other hand, the importance of educating everyone involved in pet chinchilla ownership.

Over 90% of the focus animals were allowed to move freely outside their cages or enclosures at least temporarily. This compares well with the study conducted in Italy [6], where 88.8% of the animals had access to an additional running area. The opportunity to roam freely seems essential for the welfare of the animals, especially when they are kept in small main living areas. A study conducted on a fur farm [17] found that chinchillas kept in the smallest cages with wire floors moved significantly less and rested more than those in larger cages or those with deep litter on the floor.

According to Austrian law [61], sleeping caves as well as a daily dust bath and special chinchilla bath sand must be offered. In the present study, almost all chinchillas were provided with sleeping houses at all times. However, three participants stated that they never offered one. Almost all caretakers constantly provided their focus animal a dust bath; only one focus animal was never offered a dust bath. This is consistent with the findings of the study conducted in Italy, where 0.7% of caretakers did not offer a dust bath [6].

Dust or sand also met the animals’ needs, with the exception of two participants offering sand from a hardware store. A constant offer of dust/sand baths generally carries the risk of eye inflammation and lung irritation [2], but as these illnesses were reported by only a few participants, they did not appear to constitute a general problem.

Most of the focus animals were kept in well-equipped main living areas (including climbing and hiding options), which is essential for animal welfare. A study conducted in pet rats showed that a loss of appropriate cage equipment can lead to depression-like states [63].

Running wheels were provided to almost half of the focus animals, a small proportion of which had an open running surface with crossbars (7.3%) or were made of plastic (0.6%). A study carried out in German pet stores found a risk of injury due to open running surfaces in 14.9% of the running wheels [29]. Plastic should also be avoided due to the risk of injury caused by swallowing small parts [16].

Regarding climate control, one-third of participants stated that they did not use any measuring or control devices for indoor climate in the room where chinchillas were kept. In a study conducted in Italy, 3.8% of chinchilla caretakers did not measure temperature and 48.3% did not measure humidity in the environment of the chinchillas [6]. Since a rather cool, dry climate is important for the health of chinchillas [18], caretakers should be educated about options for climate control and the potential adverse effects of heat stress and excessive humidity. However, it must be noted that our questionnaire, which was available from May until September, did not ask participants to specify the time periods when climate control measures were used, nor the minimum/maximum temperature or humidity.

In terms of feeding, hay, which is essential for chinchillas [23,25], was constantly offered by 90.1% of caretakers. In the Italian study, 86.1% of caretakers consistently offered their chinchillas hay [6]. Over 50% of participants provided commercial food at least once a day. Since commercial food was not defined in more detail in our questionnaire, we cannot identify whether it was healthy food made specifically for chinchillas or unhealthy concentrate mixtures containing high-calorie grain and easily digestible carbohydrates. Concentrate food should only be fed to a limited extent [3,15] and fruits and nuts should be avoided [26,27]. In most cases, products that were harmful to health such as dairy products, chocolate, and snacks including chips and commercial treats such as yogurt drops were never fed, except for nuts and fruits, which were offered at least once a day by more than one-third of the caretakers. Therefore, it is safe to assume that the majority of caretakers appear well informed about the nutritional needs of chinchillas but lack knowledge about not feeding them nuts and fruits.

### 4.4. Health and (Health) Care Measures

Almost 15% of focus animals were acutely or chronically ill at the time of the study, which is only about half compared to the findings in Italy at 28.1% [6]. Dental problems were reported most frequently (42.2%) and are common in chinchillas, as a study from the UK showed, in which 35% of the chinchillas examined had dental problems [64]. This may be why checking the incisors was listed as the care measure carried out most frequently.

Around two-thirds of participants stated that they presented their focus animal to veterinarians at least once a year or when necessary. However, one in eight chinchillas (12.3%) was never examined by veterinarians, which is similar to the findings (15.6%) in Italy [6]. This could mean that examination by a veterinarian was not yet necessary, or that they are not inclined to take them to a veterinarian for various reasons. Due to the fearful nature of chinchillas, some caretakers might refrain from taking them to a veterinarian for a preventive health check only. A study conducted on cat caretakers showed, for instance, that some caretakers do not take their cats to the vet because they fear the stress [65]. Similar to carrier training for cats, carrier training based on desensitization and counter-conditioning [66] might help to increase the number of caretakers taking their chinchillas to veterinarians for regular check-ups; educating caretakers about appropriate ways of transporting chinchillas might also be helpful. Another reason for refraining from taking chinchillas to a veterinarian for preventive health care might be difficulty finding a small animal practitioner specialized in chinchillas [44,67].

### 4.5. Human–Animal Relationship

Participants scored generally quite high on the Comfort from Companion Animal Scale (CCAS) [48], therefore indicating feeling rather close to their animals, with a mean score of 35.9 ± 5.5. Compared to scores for other pets (ratings with mean scores of 39.6 ± 4.8 for cats, 40.1 ± 4.8 for dogs, 39.5 ± 4.9 for ferrets, and 37.8 ± 5.3 for rats [47,48,68]), emotional closeness to chinchillas was slightly lower. One reason for this slight difference might be the shy nature of the animals, which might discourage caretakers from regular close contact and interactions that could promote even stronger attachment. For instance, carrying around was not reported as frequently as in other species, e.g., rats [68]. This is supported by the results, where a distinction was made as to whether an interaction was never carried out because the animal did not want it or because the participant did not consider it important or did not want it. Therefore, interactions such as lifting or carrying around were usually not performed as often because caretakers assumed that the animal did not want to engage in this type of interaction. One interpretation would be that caretakers know the needs of their animals, for instance, that they do not like close interactions such as being carried around and, as a result, do not want to subject their animals to unnecessary stress and forego an interaction that they may have desired. For the regression model, “never”, irrespective of the reason, was summarized into one category because, from the animals’ point of view, only frequency was relevant. Moreover, earlier studies in other species such as cats and rats only assessed the frequency of interaction (e.g., [39,68]).

The most common interactions with the chinchillas were talking (94.2%) and observing (94.2%), followed by hand-feeding (83.3%), which the majority of caretakers performed at least once per day, while half of the participants (50.4%) said they never carried the chinchillas around. This underlines their understanding of their chinchillas’ preferences. At least half of the caretakers of chinchillas seem to be aware of the fearful nature of their animals and adjust interactions, as the animals do not want to be carried around. In a survey conducted among pet rat caretakers [68], the two most common interactions were talking (98.5%) and hand-feeding (89.1%). However, more than half of rat caretakers reported carrying their rats around at least once a day, while only 14.4% of chinchilla caretakers reported doing so. Caretakers of rats and chinchillas seem to adapt to how often and how closely the animals want to interact, which is vital for a good animal–human relationship [43].

Most caretakers spent about an hour per day with their focus animal on average, which is similar to the study on pet rats, at 1.6 h [68]. There was a large variation in answers ranging from less than ten minutes to more than ten hours. It cannot be ruled out that some participants interpreted the question differently. The survey was carried out during the COVID-19 pandemic, which means that the mere presence of participants working from home or during lockdown may have been viewed as engagement with the focus animal. However, the mere presence of caretakers might also have an effect on the animals because they can habituate to humans and become tamer as a result. It should be noted that the perception of a generally positive human–animal relationship was based primarily on the caretakers’ perspective. We still found that 9.4% of chinchillas avoided their caretakers several times a day. This underscores the need for further studies to observe chinchilla behavior toward the caretaker rather than relying solely on caretaker reports.

### 4.6. Chinchilla Behavior

Most focus chinchillas showed play and affiliative behavior (e.g., cuddling/lying snuggled up with conspecifics) toward conspecifics at least several times a week. As play and affiliative behavior are primarily regarded as indicators of good welfare [69], playing, cuddling/lying snuggled up with conspecifics, and simultaneous peaceful eating can be interpreted as signs of positive welfare in most cases [70,71]. Concerning agonistic behavior, driving away/blocking conspecifics from food was most frequently mentioned, but still never occurred, according to 66% of participants. This is in line with the literature pointing out the rather peaceful nature of chinchillas and the rare occurrence of chasing, fighting, and biting, except for the introduction of new animals, separation of animals, or incorrect group composition [1,14,15].

The most frequently mentioned repetitive behavior was running up and down in a certain cage/enclosure area or jumping between two locations. Fur biting, the behavioral problem most frequently described in the literature, came in third place. Participants stated that 17.1% of focus animals showed fur biting at least once a month, which is very high compared to the study from Italy [6], in which 8.3% showed fur biting at least sometimes in the cage/enclosure and 6.3% in the running area. Studies on fur farms showed an average prevalence of fur biting in 3–5% of chinchillas, with a range of 0.0% to a maximum of 16.7% [8,10,11]. One explanation might be that caretakers misinterpret animal behavior, i.e., normal grooming behavior is considered as fur biting. The underlying reason for showing repetitive behaviors or stereotypies may be found in living areas that are too small and lacking in stimuli, animals being kept alone, or boredom [17,72].

The disliked behavior most frequently mentioned was chewing on furnishings (76.5%), which is in line with corresponding findings in Italy [6], although Italian caretakers reported it less often (40.8%). Therefore, housing conditions in which chinchillas have the entire apartment at their disposal at all times, as three participants stated, should be viewed critically. For example, the animals could endanger their lives by falling off higher furniture, nibbling cables, plants, or substances that are toxic to them if they are not supervised constantly during roaming. Even in a supervised environment, electrical cables, poisonous plants, and other hazards should be removed or protected in advance [73,74,75].

In most cases, participants rarely reported unwanted behavior directed toward them (e.g., showing aggression, biting, spraying urine). The most frequent negative reactions toward caretakers were avoiding/running away, followed by making noises (e.g., chattering of teeth) and freezing/startling. A study investigating the behavior of rodents and caretakers’ satisfaction showed that negative behaviors of chinchillas toward the caretaker were reported rarely and no aggressive behavior was reported, even though 3 of 13 chinchillas had bitten their caretakers once or twice [4]. The study also showed that caretakers, despite these bites, did not see the animals as aggressive but rather interpreted the biting incidents as play or accidents [4].

There were some potentially contradictory statements regarding the behavior of the focus animal. On the one hand, most participants stated that their focus animal was tamed using various methods and that they did not agree with the statement that their focus animal was generally stressed. On the other hand, almost two-thirds of animals reacted at least temporarily by running away, and over a third reacted by being frightened or frozen when their caretakers approached them. Participants may have misremembered the focus animal’s behavior or their own behavior or judged the animal to be tamer or more relaxed than it actually was. Another reason could be that they had a different understanding of the word “tame” and might take the generally shy nature of chinchillas into consideration. Caretakers who do not spend intensive periods of time with their chinchillas may also not recognize or may misinterpret behavior indicative of stress. Since we did not ask directly/specifically how many focus animals were perceived as tame, but only how they were tamed and about various reactions to caretakers, this factor cannot be directly compared, and further investigations are needed.

### 4.7. Associations with the Perceived Stress Level of the Focus Animal

In our study, caretakers perceived their chinchillas as generally more stressed if the focus animal was currently ill. The fact that there exists a connection between stress and illness has already been proven in other studies (reviewed by [76,77]). Participants also stated higher perceived stress levels in their animals when they showed more fearful behavior towards the caretaker. Caretakers might interpret fearful behavior as stress responses. For fur biting, only a trend for higher perceived stress in the case of a higher frequency of fur biting was found. Stress can promote repetitive behaviors [78]. Fur biting, which is sometimes interpreted as repetitive behavior, may also be caused or enhanced by stress [7,9,11]. However, as fur biting may not always occur in the presence of caretakers, it might be under-reported, which could explain the lack of a significant association. If fur biting is shown weakly or infrequently, caretakers may simply not recognize it. Studies of cats and dogs [79,80] have shown that only conspicuous, potentially disruptive, or frequent behaviors were perceived by caretakers as signs of stress.

Perceived stress levels also tended to be higher when smaller numbers of chinchillas were kept together and when affiliative behaviors were observed less frequently. The most likely explanation is mechanisms of social buffering of stress [81]. It cannot be ruled out that signs of stress in the focus animal could be better observed and perceived if fewer chinchillas were kept together in the same cage/enclosure. However, it is more likely that higher rates of affiliative behaviors counteracted stress responses. The larger the number of conspecifics, the greater the likelihood of affiliative behaviors observed. It must be pointed out that individually kept animals were not included in this regression model because we were interested in associations with social behavior. However, compared to pair and group housing, the number of animals kept individually was low and would have drastically decreased the sample size for the model.

Agonistic and repetitive behavior (e.g., “running up and down or jumping at a certain place in the cage/enclosure or between two places”, which could be interpreted as potential escape attempts), did not relate significantly to perceived stress levels. This could be due to the relatively low occurrence or observation of agonistic and repetitive behaviors. Another reason could be that caretakers, for instance, interpreted the attempt to escape as a normal urge to move and did not classify it as “running up and down or jumping at a certain place in the cage/enclosure or between two places”, which we classified as repetitive behavior. Attempts to escape in case of unsuitable conditions can develop into stereotypies [82,83].

### 4.8. Associations with Emotional Closeness to Focus Animals

The model showed that caretakers who spent more time engaging with their focus animals also felt emotionally closer to them. This is in line with findings of other studies demonstrating that caretakers’ attachment to their pet relates positively to the time spent with the animal (e.g., in ferrets, dogs, cats [47,84,85,86]). Fearful and agonistic behavior toward the caretaker did not relate to emotional closeness to the animal. It is possible that these behaviors are considered normal for chinchillas by caretakers as, despite the fact that chinchillas have been kept and bred in captivity over generations, they are generally considered rather shy and nervous animals [15,19].

Moreover, the occurrence of disliked behavior did not relate significantly to emotional closeness. However, the occurrence of such behavior was in general low. Another reason could be that caretakers are quite tolerant of unwanted behaviors and are ready to classify bite incidents, for instance, as play or an “accident” [4], as mentioned above.

What the model also showed was that caretakers tended to be more emotionally close to their chinchillas when there were no children younger than twelve years in the household. This is consistent with findings from other studies, e.g., in cats [40,41], and comes as no surprise as pet caretakers without responsibility for children may have more time and general capacity available for their animals. Also, some participants may consider their animals as a substitute or expect them to prevent loneliness [87]. Moreover, oxytocin as a “bonding hormone” creates close bonds both between parents and children, and pet caretakers and their animals [88,89].

### 4.9. Limitations of the Study

Using an online questionnaire has advantages and disadvantages over other methods. Caretakers can provide a very valuable insight into their animals’ behavior. In addition, it is inexpensive, allows one to gain knowledge of behaviors that are difficult or very time-consuming to study, requires relatively little time for data collection, and can reach a large number of potential participants (reviewed by [90,91]). The disadvantages are that there can be biased answers or the animal caretaker’s assessment of the chinchilla’s behaviors is unclear. There may be some subjectivity in how caretakers interpret their animal’s behavior, but they know their animals best, based on their daily contact. Furthermore, subjective biases are also possible in the self-description of human behavior because the participants’ answers might be affected by aspects of social desirability and they might not remember events correctly (e.g., exaggeration or understatement, both consciously and subconsciously) [90,92,93]. While using a questionnaire excluded the possibility of observing animal behavior, this could be part of a follow-up study to get more insight on specific aspects that were not covered in this study or to supplement the findings presented with observation.

It must be pointed out that the analyses are based on a self-selected sample of German-speaking, mainly female, chinchilla caretakers responding to an online questionnaire. As participation was voluntary, interested and motivated caretakers were more likely to take part in the study, which may be why few welfare issues were identified. Hence, our sample might not be representative of all chinchilla caretakers or caretakers in other countries.

Finally, due to the nature of the study, only conclusions on associations, not on causal relationships, can be drawn.

## 5. Conclusions

Our study provides an extensive overview of the housing conditions in which pet chinchillas are kept, with a focus on welfare aspects. On the one hand, the results showed that the conditions in which the chinchillas were kept varied, but that the most essential needs, such as keeping them with other chinchillas, availability of a dust bath and elevated areas, and constant access to water and hay, were met in most cases. On the other hand, there is a need for improvement or clarification, especially with regard to cage/enclosure size, individual housing, and climate (monitoring) in the room where the chinchillas are kept. While many chinchillas showed behavioral indicators of good welfare, including playing or affiliative behavior, behavioral indicators of impaired welfare, such as biting conspecifics or backflips, were rare.

Additionally, education and training could help caretakers to better assess and “read” their animals, as the study suggests that caretakers do recognize stress in their focus chinchilla if the animal is currently ill or shows fearful behavior, but not necessarily when abnormal/repetitive behaviors and agonistic behaviors are displayed. Since the emotional closeness between caretakers and their focus chinchillas was generally quite high, this indicates a positive human–chinchilla relationship from the caretakers’ perspective in most cases. Our results contribute to a better understanding of husbandry conditions and behavior of pet chinchillas. Follow-up studies should validate our results using observational methods.

## Figures and Tables

**Table 1:** Description of questionnaire sections.

Section	Overview of Questions Relevant to the Study
General information on participants and chinchilla-keeping	Previous experience with chinchilla husbandry, number of chinchillas currently owned, purpose of keeping, time spent with all chinchillas each day
General information on focus chinchilla	Origin, sex, neuter status, age, duration of keeping
Social management, housing, equipment and enrichment, roaming opportunities	Group composition (number and sex of animals in same housing system), reason for solitary keeping, group size change, type and size of and amount of levels in current housing system, access to and time spent in additional running area, systems to control indoor climate, access for other pets, availability of cage equipment, material and running surface of running wheel, access to dust bath and type of sand/litter
Nutrition	Frequency of access to different types of food
Health of focus chinchilla	Current illnesses
Care and maintenance activities	Grooming routine, frequency of vet visits, frequency of cleaning housing system
Behavior of focus chinchilla	Frequency of occurrence of specific behaviors of the focus animal within the last month, focusing on behavior potentially indicative of impaired welfare (e.g., biting/nibbling own fur and other repetitive or unwanted behaviors), frequency of selected behaviors toward conspecifics (e.g., playing or biting), frequency of selected behaviors towards caretaker (e.g., avoiding/running away)
Human–animal relationship	Time spent with focus animal each day, method of taming, attachment to the focus chinchilla (using the ‘Comfort from Companion Animal Scale’ by [48], frequency of human–animal interactions (e.g., talking to the animal, training the animal)
Stress perception	“Perception of whether the focus animal is generally stressed” (answered on a 7-point Likert scale, from ‘completely agree’ to ‘completely disagree’; referred to as “perceived stress level” in the manuscript for the sake of simplicity)
Demographic data	Details about the respondent (gender, age), number of people living in the household, presence of children living in the household, place of residence (country)

**Table 2:** Characteristics of participants and households.

Characteristics; *n*	Number of Participants	Percentage of Participants (%)
Place of residence (country); *n* = 302		
Austria	38	12.6
Germany	253	83.8
Luxembourg	10	3.3
Switzerland	1	0.3
Age of participant (years); *n* = 300		
18–25	59	19.7
26–33	89	29.7
34–41	62	20.7
42–49	49	16.3
50–57	27	9.0
58–66	14	4.7
Number of people living in the household; *n* = 300		
1	49	16.3
2–3	209	69.7
4–5	38	12.7
6–9	4	1.3
Children living in the household; *n* = 301		
Children aged 0–11 years present	45	15.0
Children aged 12–16 years present	37	12.3
Previous experience with chinchilla husbandry (in years); *n* = 334		
< 1	27	8.1
1–2.5	67	20.1
3–5.5	50	15.0
6–10	70	21.0
11–15	33	9.9
>15	87	26.0
Number of current chinchillas owned; *n* = 336		
1	14	4.2
2	161	48.0
3	62	18.5
4–5	56	16.7
6–10	20	6.0
11–20	9	2.7
21–60	12	3.6
>100	2	0.6
Purpose of keeping (multiple answers possible); *n* = 336		
As a pet (for participants themselves)	323	96.1
As a pet for children	19	5.7
Keeping animals for breeding as a hobby	17	5.1
Keeping animals for professional breeding	7	2.1
Other	6	1.8

**Table 3:** Characteristics of focus chinchillas and contact with other animals.

Focus Animal Characteristics, Group Composition, Contact with Other Species	Number of Focus Animals	Percentage (%) of Focus Animals
Sex and neuter status; *n* = 332		
Female neutered	5	1.5
Female unneutered	152	45.8
Female neuter status unknown	3	0.9
Male neutered	56	16.9
Male unneutered	115	34.6
Male neuter status unknown	1	0.3
Group composition (without single keeping); *n* = 258		
Female pair	36	17.1
Male pair	47	22.4
Mixed-sex pair	59	28.1
All female group of three or more	16	7.6
All male group of three or more	16	7.6
Mixed-sex group of three or more	36	17.1
Contact with other animals (multiple answers possible); *n* = 329		
None	216	65.7
Contact with dogs	67	20.4
Contact with cats	53	16.1
Contact with other animals	19	5.8

**Table 4:** Overview of how often participants reportedly offered different furnishings and enrichment in the main living areas (constant or almost always accessible area). The higher the percentage, the darker the shade of blue.

Furnishings and Enrichment	*n*	Frequencies (%)						
		Constant	>1/Day	1x/Day	>1/Week	1x/Week	<1x/Week	Never
Boards at different heights	335	99.1	0.0	0.3	0.0	0.0	0.3	0.3
Movable roots, sticks, or branches	334	85.9	1.2	1.2	4.2	2.1	1.8	3.6
Fixed roots, sticks, or branches	331	80.7	0.3	0.9	0.9	0.6	1.8	14.8
Swings/Hammocks	326	56.1	0.3	0.3	0.6	0.3	2.1	40.2
Houses	335	98.2	0.3	0.3	0.0	0.0	0.3	0.9
Tunnels/Tubes	333	94.6	0.3	0.3	0.9	0.0	1.2	2.7
Ladders/Stairs	329	66.6	0.0	0.3	1.2	0.3	0.0	31.6
Bridges	328	71.3	0.6	0.6	0.9	0.3	1.5	24.7
Cardboard boxes	323	13.0	1.5	3.4	5.6	4.3	14.9	57.3

**Table 5:** Feeding furnishings, water provision, and frequency of feeding different products. The higher the percentage, the darker the shade of blue.

Equipment and Feeding Routines	*n*	Frequencies (%)						
		Constant	>1/Day	Daily	>1/Week	1 x/Week	<1/Week	Never
Food bowls	334	95.2	0.6	3.3	0.6	0.3	0	0
Water sources (water bowls, nipple drinkers)	334	98.2	0.3	0.9	0.6	0	0	0
Carpet out of hay	302	37.1	0.3	0.3	2.3	0.7	1.3	55.3
Hayrack	322	74.2	0.3	1.2	1.2	1.2	0.6	21.1
Food tree/food ball	326	39.3	1.2	4.3	14.1	10.4	11.7	19
Commercial food for chinchillas	333	38.4	1.2	20.7	9.6	5.4	3.6	21
Hay	333	90.1	0.6	4.2	2.7	0.9	0.6	0.9
Grass	322	13.7	0.9	3.7	12.1	4	10.9	54.7
Vegetables (fresh or dried)	332	14.5	3	14.5	28	14.8	15.1	10.2
Fruits (fresh or dried)	331	9.1	2.7	14.5	28.1	16.9	20.8	7.9
Herbs or leaves (fresh or dried)	333	76	3.3	10.5	6.6	1.8	1.2	0.6
Salt lick	327	15.3	0.3	0.9	0.3	0.6	2.8	79.8
Nuts or kernel	334	3.6	3.6	29.6	25.4	12.3	14.1	11.4
Dairy products	330	0	0	0	0	0	0	100
Commercial treats, e.g., yogurt drops	331	0	0.9	2.4	3.6	3	9.7	80.4
Hard bread	330	0	0	0	0.3	1.2	5.5	93
Vitamin supplements or mineral supplements	330	1.5	0	2.7	1.2	4.2	14.5	75.8
Chocolate or snacks	330	0	0	0	0	0.3	0.3	99.4

**Table 6:** Overview of cleaning routines and frequency of health checks and care measures performed by caretakers. The higher the percentage, the darker the shade of blue.

Cleaning Routines and (Health) Care Measures	*n*	Frequencies (%)							
		>1x/ Day	1x/ Day	>1x/ Week	1x/ Week	>1x/ Month	1x/ Month	<1x/ Month	Never
Cleaning whole enclosure	328	1.5	19.8	14.6	36.3	13.1	13.4	1.2	0
Cleaning running area	318	3.1	34.6	25.5	22	5	4.7	0.6	4.4
Changing litter in enclosure only	323	0.9	18	23.8	24.1	10.2	5.9	1.5	15.5
Changing of sand/litter in dust bath only	322	0.9	18.3	22	28.3	11.5	9.6	2.8	6.5
Control of the ears	324	0.3	13	8	25.9	8.3	19.8	16.4	8.3
Control of incisors	327	0.3	4	3.4	22.9	9.2	28.7	23.9	7.6
Control of anal area	326	0.3	11.7	9.2	21.8	12	17.2	18.7	9.2
Grooming	327	0.3	4.6	2.8	8.3	5.5	5.5	11	62.1
Cleaning around eyes	324	0.6	4	4.3	6.5	3.4	4.9	13	63.3
Cleaning around nose	324	0.3	2.2	0.9	4.9	2.5	1.9	14.2	73.1

**Table 7:** Overview of frequency of various human–animal interactions reported by caretakers. The higher the percentage, the darker the shade of blue.

Human–Animal Interactions	*n*	Frequencies (%)								
		>1x/ Day	1x/ Day	>1x/ Week	1x/ Week	>1x/ Month	1x/ Month	<1x/ Month	Never Because I Do Not Want To/Do Not Think It Is Necessary	Never Because Focus Chinchilla Does Not Like It
Observing	310	75.8	18.4	3.9	0.6	0.3	0.0	0.0	0.3	0.6
Lifting up	312	11.2	18.6	12.2	9.3	8.3	5.8	8.3	5.4	20.8
Carrying around	311	6.4	8.0	8.7	4.5	2.3	2.3	4.5	14.5	48.9
Talking to	312	79.5	14.7	3.8	0.6	0.0	0.0	0.6	0.6	0.0
Clicker training	301	2.3	1.3	2.0	0.7	0.3	0.7	4.7	70.8	17.3
Target training	293	1.4	1.0	1.4	2.0	0.7	1.7	3.8	68.9	19.1
Hand-feeding	312	45.8	37.5	11.9	1.0	1.3	0.6	0.3	0.3	1.3

**Table 8:** Frequency of behaviors displayed by focus animals in the last month as reported by caretakers. The higher the percentage, the darker the shade of blue.

Behavior of Focus Animal	*n*	>1/ Day	1x/ Day	>1/ Week	1x/ Week	>1/ Month	1x/ Month	Never
Behavior toward conspecifics								
Biting/nibbling fur of conspecifics	279	8.2	2.2	9.3	0.7	3.2	3.9	72.4
Biting of conspecifics	279	0.0	0.7	0.7	0.0	0.7	2.9	95.0
Hunting or fighting with conspecifics	280	0.0	1.1	2.5	0.7	4.6	15.7	75.4
Playing with conspecifics	276	40.9	8.0	19.9	4.3	10.9	0.7	15.2
Driving away or blocking conspecifics from food	281	3.6	2.1	7.8	3.9	10.0	7.1	65.5
Spraying conspecifics with urine	278	0.0	0.4	0.0	0.7	2.5	6.1	90.3
Cuddling/lying snuggled up with conspecifics	281	87.9	3.6	6.0	0.7	0.7	0.0	1.1
Simultaneous, peaceful eating with conspecifics	281	82.2	6.8	7.1	0.4	2.1	0.0	1.4
Behavior toward caretaker								
Avoiding/running away	308	9.4	8.8	16.9	5.8	13.6	7.1	38.3
Freezing/startling	306	2.9	3.6	6.9	2.9	11.4	11.4	60.8
Making noises (e.g., chattering of teeth, hissing, screaming, single shrill sounds)	309	5.5	2.3	7.1	5.8	11.7	15.5	52.1
Interacting negatively (e.g., showing aggression, spraying urine, biting)	309	0.0	0.0	0.3	0.3	0.6	2.9	95.8
Unwanted and repetitive behavior								
Biting/nibbling own fur	304	5.9	1.0	4.9	1.3	2.3	1.6	82.9
Bar biting/bar shaking	307	0.7	4.2	4.2	2.9	5.9	7.2	74.9
Backflips	306	1.6	0.0	1.0	0.0	1.3	1.3	94.8
Running up and down or jumping at a certain place in the cage/enclosure or between two places	306	8.8	2.6	5.9	2.3	8.2	2.3	69.9
Quick spinning	307	2.0	0.3	2.0	0.7	0.7	1.3	93.2
Biting into own tail	306	0.0	0.0	0.0	0.0	1.3	0.3	98.4

**Table 9:** Linear regression models of perceived stress levels (*n* = 191) and emotional closeness to chinchillas, assessed by means of the Comfort from Companion Animal Scale (CCAS) (*n* = 185). Differing *n* is due to incomplete datasets.

Dependent Variables	Independent Variables and Model Summary	Estimate ^a^	SE ^b^	Beta ^c^	t	*p*
Level of perceived stress	Constant	2.79	1.09		2.56	0.011
	Frequency of dust bath access	−0.19	0.12	−0.11	−1.58	0.116
	Time in running area	0.02	0.03	0.04	0.59	0.558
	Frequency of running area access	0.23	0.17	0.10	1.35	0.179
	Size of main living area	0.00	0.03	0.01	0.15	0.884
	**Number of chinchillas kept together (with focus animal) in main living area**	−0.18	0.10	−0.13	−1.84	***0.067***
	Daily time spent with focus animal	−0.06	0.06	−0.08	−1.01	0.313
	**Current illnesses**	0.45	0.21	0.15	2.09	**0.038**
	Disliked behavior	0.25	0.21	0.09	1.22	0.226
	**Fur biting**	0.09	0.05	0.14	1.86	***0.065***
	Repetitive behavior/escape attempts	−0.07	0.06	−0.09	−1.18	0.238
	Other abnormal repetitive behavior	−0.01	0.16	−0.01	−0.09	0.930
	Agonistic behavior	0.02	0.12	0.01	0.13	0.897
	**Affiliative behavior**	−0.11	0.07	−0.12	−1.71	***0.090***
	**Fearful behavior in presence of caretaker**	0.15	0.06	0.21	2.67	**0.008**
	Agonistic behavior toward caretaker	0.49	0.39	0.09	1.25	0.213
	Model: adj. R^2^ = 0.14, F = 3.01, *p* < 0. 001					
CCAS	Constant	22.51	8.21		2.74	0.007
	**Children (up to 12 years) in household**	−1.68	1.01	−0.12	−1.67	***0.096***
	Time in running area	−0.22	0.14	−0.12	−1.54	0.126
	Frequency of running area access	0.69	0.86	0.06	0.81	0.422
	Frequency of dust bath access	0.17	0.63	0.02	0.27	0.789
	**Daily time spent with focus animal**	0.91	0.36	0.21	2.51	**0.013**
	Frequency of health checks	0.31	0.27	0.09	1.15	0.254
	Frequency of (health) care	0.37	0.28	0.10	1.31	0.191
	Frequency of ‘observe talk hand-feed’	0.46	0.83	0.04	0.55	0.585
	**Frequency of lifting and carrying around**	0.26	0.14	0.14	1.84	***0.068***
	Frequency training	0.27	0.28	0.07	0.96	0.340
	Disliked behavior	0.52	1.03	0.04	0.50	0.618
	Agonistic behavior	−0.93	0.64	−0.11	−1.45	0.149
	Affiliative behavior	0.26	0.33	0.06	0.80	0.423
	Fearful behavior in presence of caretaker	0.22	0.28	0.06	0.79	0.433
	Agonistic behavior toward caretaker	2.34	1.88	0.09	1.25	0.215
	Model: adj. R^2^ = 0.12, F = 2.71, *p* < 0. 001					

^a^ Estimate: estimated regression coefficient; ^b^ SE: Standard error of estimate; ^c^ Beta: standardized regression coefficient. Significant (*p* ≤ 0.05) independent variables are depicted in bold and trends (*p* > 0.05 ≤ 0.1) are depicted in bold and italics.

## Data Availability

Restrictions apply to the datasets. The datasets presented in this article are not readily available because they must not be shared with third parties and because of ongoing data analysis for follow-up manuscripts. Requests to access the datasets should be directed to I. Windschnurer, ines.windschnurer@vetmeduni.ac.at.

## Supplementary Materials

The following supporting information can be downloaded at: https://www.mdpi.com/article/10.3390/ani14213155/s1: Table S1: Principal component analysis of (health) care measures; Table S2: Principal component analysis of attachment; Table S3: Principal component analysis of human–animal interactions; Table S4: Principal component analysis of social behaviors; Table S5: Principal component analysis of negative behaviors in the presence of the caretakers; Table S6: Principal component analysis of unwanted and repetitive behaviors. Figure S1. Emotional closeness to the chinchillas of 299 participants, assessed by means of the Comfort from Companion Animal Scale [49]. A minimum score of eleven and a maximum score of 44 are possible, with higher scores indicating a closer attachment to the animal.
