# Supplementary material for: Husbandry Conditions and Welfare State of Pet Chinchillas (Chinchilla lanigera) and Caretakers’ Perceptions of Stress and Emotional Closeness to Their Animals
Source: Animals (Basel). 2024 Nov 3;14(21):3155. doi: 10.3390/ani14213155 (PMC11544953; doi:10.3390/ani14213155)
Supplement: Supplementary file 1 [file animals-14-03155-s001.zip › Supplementary Material_021124.pdf]

Supplementary Material

Husbandry conditions and welfare state of pet chinchillas (*Chinchilla lanigera*) and caretakers' perceptions of stress and emotional closeness to their animals

Elisabeth M. Gilhofer[1], Denise V. Hebesberger[1], Susanne Waiblinger[1], Frank Künzel[2], Cornelia Rouha-Mülleder[3], Chiara Mariti[4], and Ines Windschnurer[1,*]

[1]*Centre for Animal Nutrition and Welfare, Clinical Department for Farm Animals and Food System Science, University of Veterinary Medicine Vienna, Veterinärplatz 1, 1210 Vienna, Austria;* lisagilhofer@yahoo.com, denise.hebesberger@gmx.at, susanne.waiblinger@vetmeduni.ac.at, ines.windschnurer@vetmeduni.ac.at

[2]*Clinical Centre for Small Animal Health and Research, Clinical Department for Small Animals and Horses, University of Veterinary Medicine Vienna, Veterinärplatz 1, 1210 Vienna, Austria;* frank.kuenzel@vetmeduni.ac.at

[3]*Animal Welfare Ombudsman Office, Office of the Provincial Government of Upper Austria, Bahnhofsplatz 1, 4021 Linz, Austria;* tierschutzombudsstelle@ooe.gv.at

[4]*Department of Veterinary Science, University of Pisa, Viale delle Piagge 2, 56124 Pisa, Italy*; chiara.mariti@unipi.it

*Correspondence: ines.windschnurer@vetmeduni.ac.at; Tel: 0043 1 25077 4915

Table S1. Principal component analysis of (health) care measures (n = 321).

**(Health) care measures**	**Components and loadings**	
	**Health checks**	**Cleaning & fur care**
Ear check	0.86	
Check of anal region	0.86	
Check of incisors	0.84	
Cleaning nasal area		0.90
Cleaning eye area		0.87
Fur care (e.g. brushing)		0.59
Variance explained by component (%)	37.78	33.09
Cronbach α	0.84	0.72

Extraction method: principal component analysis.

Rotation method: Varimax rotation. Loadings below 0.3 are not shown.

Table S2. Principal component analysis of attachment. 1 = strongly disagree, 4 = strongly agree (n = 299).

**Questionnaire items**	**Component loadings**
My chinchilla is a source of constancy in my life	0.82
My chinchilla makes me feel needed	0.77
Having my chinchilla gives me something to love	0.77
My chinchilla makes me feel loved	0.75
Having a chinchilla gives me something to care for	0.74
My chinchilla makes me feel trusted	0.70
My chinchilla provides me with pleasurable activity	0.70
I get comfort from touching my chinchilla	0.69
My chinchilla provides me with companionship	0.69
My chinchilla makes me laugh and play	0.58
I enjoy watching my chinchilla	0.42
Variance explained by component (%)	49.4
Cronbach α	0.89

Extraction method: principal component analysis.

Loadings below 0.3 are not shown.

Table S3. Principal component analysis of human-animal interactions (n = 291).

**Human-animal interactions**	**Components and loadings**		
	**Observe talk hand-feed**	**Lifting and carrying around**	**Training**
Talking	0.85		
Observing	0.82		
Hand feeding	0.64		
Lifting up		0.90	
Carrying around		0.90	
Clicker training			0.86
Target training			0.77
Variance explained by component (%)	25.97	23.96	20.24
Cronbach α	0.61	0.80	0.56

Extraction method: principal component analysis.

Rotation method: Varimax rotation. Loadings below 0.3 are not shown.

Table S4. Principal component analysis of social behaviors (n = 271).

**Social behaviors**	**Components and loadings**	
	**Agonistic behaviors**	**Affiliative behaviors**
Chasing of or fighting with conspecifics	0.76	
Biting of conspecifics	0.72	
Driving away/blocking conspecifics from food	0.65	
Spraying conspecifics with urine	0.62	
Cuddling/lying snuggled up with conspecifics		0.85
Simultaneous peaceful eating with conspecifics		0.82
Playing with conspecifics		0.58
Variance explained by component (%)	28.14	25.01
Cronbach α	0.50	0.54

Extraction method: principal component analysis.

Rotation method: Varimax rotation. Loadings below 0.3 are not shown.

Table S5. Principal component analysis of negative behaviors in the presence of the caretakers (n = 304).

**Behaviors in presence of caretaker: Chinchilla…**	**Components and loadings**	
	**Fearful behaviors in presence of caretaker**	**Agonistic behaviors towards caretaker**
… freezes/startles	0.81	
… avoids me or runs away from me	0.77	
… makes noises (e.g. chattering of teeth, hissing, screaming, single shrill sounds)	0.66	
… interacts with me negatively (e.g. shows aggression, sprays urine, bites)		0.98
Variance explained by component (%)	42.29	25.75
Cronbach α	0.61	

Extraction method: principal component analysis.

Rotation method: Varimax rotation. Loadings below 0.3 are not shown.

Table S6. Principal component analysis of unwanted and repetitive behaviors (n = 273).

**Behaviors**	**Components and loadings**		
	**Fur biting**	**Repetitive behavior/escape attempts**	**Other abnormal repetitive behavior**
Biting/nibbling fur of conspecifics	0.89		
Biting/nibbling own fur	0.86		
Bar biting/bar shaking		0.77	
Running up and down or jumping at a certain place in the cage/enclosure or between two places		0.77	
Biting into own tail			0.75
Backflips			0.70
Variance explained by component (%)	26.39	21.67	17.72
Cronbach α	0.73	0.45	0.04

Extraction method: principal component analysis.

Rotation method: Varimax rotation. Loadings below 0.3 are not shown.

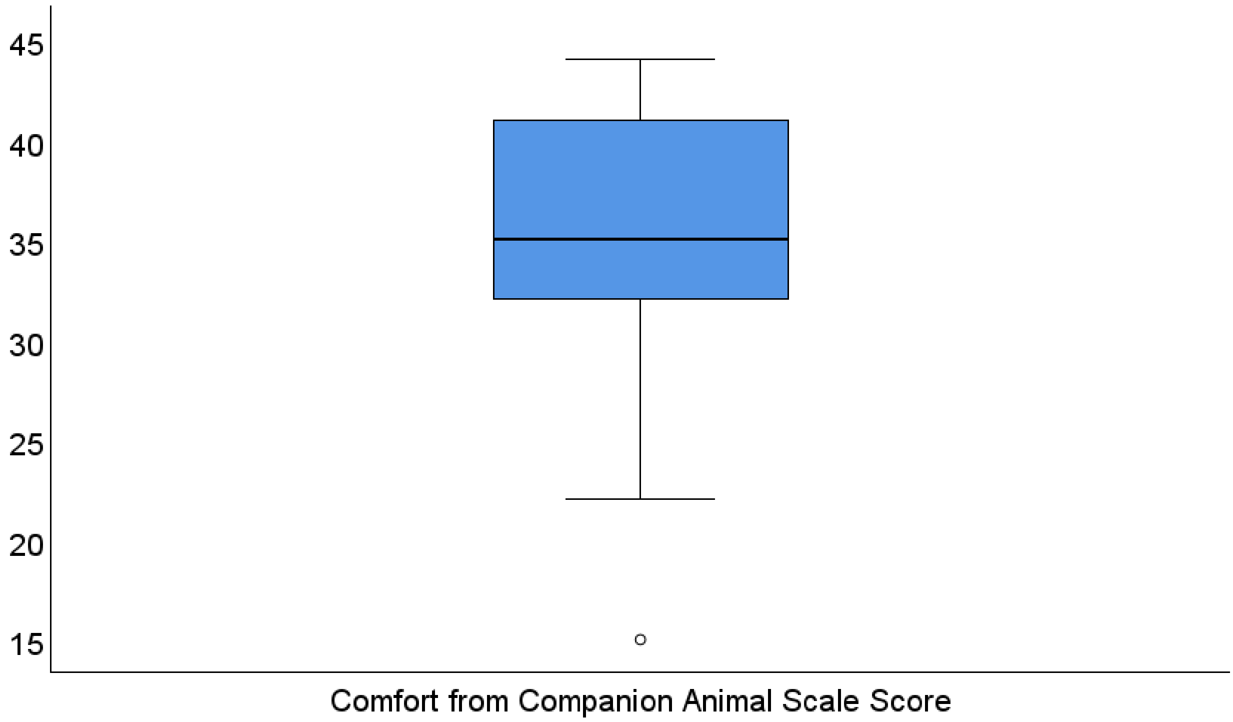

Figure S1. Emotional closeness to the chinchillas of 299 participants, assessed by means of the *Comfort from Companion Animal Scale*[49]. A minimum score of eleven and a maximum score of 44 are possible, with higher scores indicating a closer attachment to the animal.
